# Atmospheric Pressure Plasma Treatment for Grey Cotton Knitted Fabric

**DOI:** 10.3390/polym10010053

**Published:** 2018-01-08

**Authors:** Chi-wai Kan, Chui-fung Lam

**Affiliations:** Institute of Textiles and Clothing, The Hong Kong Polytechnic University, Hung Hom, Kowloon, Hong Kong, China; 11901599R@connect.polyu.hk

**Keywords:** atmospheric plasma, yellowness, dry preparation, grey cotton, water absorption, impurities

## Abstract

100% grey cotton knitted fabric contains impurities and yellowness and needs to be prepared for processing to make it suitable for coloration and finishing. Therefore, conventionally 100% grey cotton knitted fabric undergoes a process of scouring and bleaching, which involves the use of large amounts of water and chemicals, in order to remove impurities and yellowness. Due to increased environmental awareness, pursuing a reduction of water and chemicals is a current trend in textile processing. In this study, we explore the possibility of using atmospheric pressure plasma as a dry process to treat 100% grey cotton knitted fabric (single jersey and interlock) before processing. Experimental results reveal that atmospheric pressure plasma treatment can effectively remove impurities from 100% grey cotton knitted fabrics and significantly improve its water absorption property. On the other hand, if 100% grey cotton knitted fabrics are pretreated with plasma and then undergo a normal scouring process, the treatment time is reduced. In addition, the surface morphological and chemical changes in plasma-treated fabrics were studied and compared with the conventionally treated fabrics using scanning electron microscope (SEM), Fourier-transform infrared spectroscopy-attenuated total reflection (FTIR-ATR) and X-ray photoelectron spectroscopy (XPS). The decrease in carbon content, as shown in XPS, reveal the removal of surface impurities. The oxygen-to-carbon (O/C) ratios of the plasma treated knitted fabrics reveal enhanced hydrophilicity.

## 1. Introduction

Cotton is an important natural fibre for the textile and fashion industries. Cotton is essentially a cellulose which contains a large amount of polar hydroxyl groups that impart good hydrophilicity to fibres, leading to excellent wettability. Generally speaking, when cotton fabrics are in grey form, they contain natural and added impurities such as natural oil, waxes, pectin and coloring matter, etc., i.e., impurities coming from the fibre itself, the manufacturing process, as well as the environment, such as stains of machine oil and dust [1,2]. These impurities reduce the wettability of cotton fabrics. Therefore, these impurities need to be removed for subsequent dyeing and finishing processes. As most of these impurities are water-insoluble and are very difficult to remove with water alone, various types of chemicals and reagents are required to remove them.

The conventional pretreatment of 100% grey cotton knitted fabric consists mainly of scouring and bleaching, which leads to good wettability. However, the conventional pretreatment process consumes large amounts of water, chemicals and energy, and generates much waste water which has high chemical oxygen demand (COD) and biological oxygen demand (BOD) values [1,2,3,4,5]. Because of increasing environmental concerns, a cost-effective and environment-friendly pretreatment process is desirable as an alternative to the conventional pretreatment process.

In the past few decades, plasma treatment has been developed and it has the capability for altering textile material surfaces without affecting their bulk properties [6,7,8,9,10,11,12,13]. The plasma process is entirely non-aqueous and, depending on the plasma species used, different types of surface modification of textile materials can be achieved [14,15,16,17]. In addition, plasma treatment can change the polymer solubility behavior [16,18] such that it can be used for surface cleaning. As mentioned, grey cotton fabric contains a lot of natural and added impurities which are high molecular weight substances. Therefore, in this study, we investigate the possibility of using atmospheric pressure plasma as a dry pretreatment process to remove impurities from 100% grey cotton knitted fabric. If the wettability of the plasma treated fabric is improved, it can be an alternative to the conventional pretreatment process.

## 2. Experimental

### 2.1. Materials

Grey single jersey and interlock fabrics (100% cotton) were used in this study, having specifications as shown in Table 1.

### 2.2. Conventional Preparation Processes

#### 2.2.1. Cotton Scouring

The cotton knitted fabrics were scoured with sodium hydroxide (30 g/L), non-ionic detergent (2 g/L) and sodium silicate (2 g/L) (supplied by Tin Hang Technology Limited, Hong Kong, China) with a liquor-to-goods ratio of 50:1 at 100 °C for 60 min. After scouring, the fabric specimens were washed with 0.5% sulfuric acid for neutralisation. Then, the specimens were washed with water at 30 °C for 5 min and completely dried in atmospheric conditions.

#### 2.2.2. Cotton Bleaching

Cotton knitted fabrics were bleached with hydrogen peroxide (17.5 g/L), Sandozin NI (1 g/L), sodium hydroxide (5 g/L), sodium silicate (10 g/L) and magnesium sulfate (5 g/L) (supplied by Tin Hang Technology Limited, Hong Kong, China) with a liquor-to-goods ratio of 50:1 at 90 °C for 60 min. The pH of the bleaching solution was adjusted to 11 by sodium hydroxide. After bleaching, the specimens were washed with water at 30 °C for 5 min and then dried under atmospheric conditions.

### 2.3. Atmospheric Pressure Plasma Treatment

**Figure 1:**
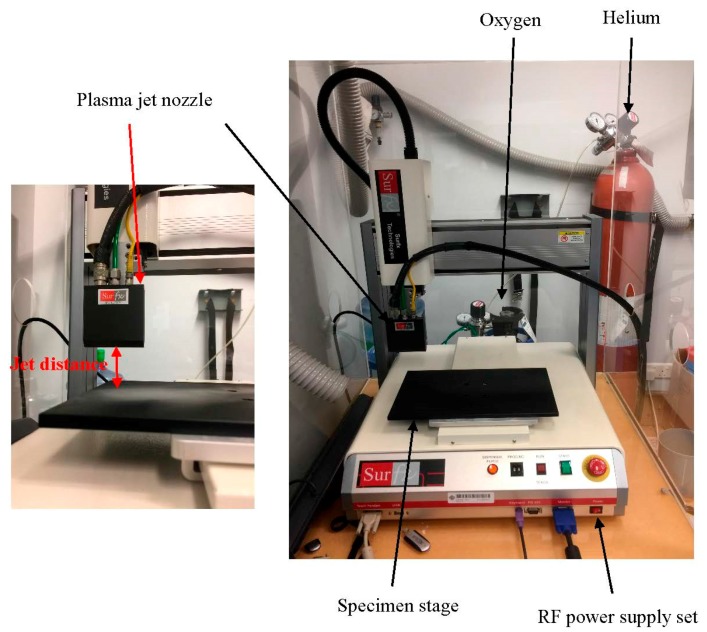
Set-up of the atmospheric pressure plasma jet (APPJ).

**Figure 2:**
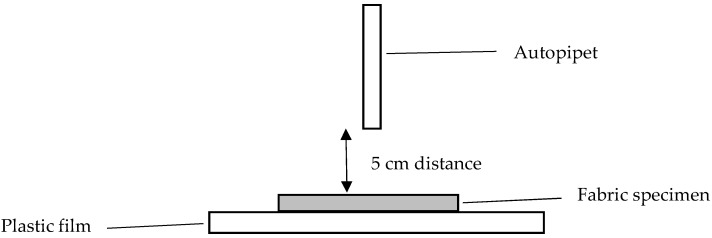
Drop test.

The atmospheric pressure plasma treatment was conducted with an atmospheric pressure plasma jet (APPJ) system (Model: Atomflo 200-Series) manufactured by Surfx Technologies (Redondo Beach, CA, USA). The APPJ system has a one-inch linear-beam head connected to the gas supply. Helium (purity ≥99.995%) and oxygen (≥99.7%) (Hong Kong Oxygen, Hong Kong, China) were used in the plasma treatment (Helium is used as carrying gas and for facilitating ionisation of oxygen). The set-up and schematic diagram of the APPJ used in this study are illustrated in Figure 1 and Figure 2, respectively.

Specimens of size 15 cm × 15 cm were conditioned in a standard testing environment (20 ± 2 °C, 65% ± 2% Relative humidity (RH)) for 24 ± 1 h before plasma treatment. After weighing, the fabric was laid flat and fixed well in a square aluminum frame without creases. Then, the specimen was placed under the nozzle of the APPJ for plasma treatment. The nozzle was mounted vertically above the fabric specimen and the active area of the nozzle was 1 × 25 mm^2^. The nozzle directs a plasma beam (generated by radio-frequency (13.56 MHz) on to the specimen and scans its surface. The plasma treatment was operated according to the parameters summarised in Table 2. In the plasma treatment, when one parameter was changed, the other parameters were held constant. After plasma treatment, treated fabric specimens were conditioned under standard environmental conditions (20 ± 2 °C, 65% ± 2% RH) for 24 ± 1 h prior to evaluation tests.

### 2.4. Weight-Loss Measurement

In order to determine the amount of impurities removed by the treatment, cotton knitted fabrics were weighed before and after the treatment [19,20]. The weight of fabric was measured before (*W*_0_) and after (*W*_1_) different treatments for comparison. Three measurements of weight were recorded and averaged. The percentage weight loss was calculated by Equation (1):

(1) $$\mathrm{Weight}\;\mathrm{loss}\;\left(\%\right)=\frac{W_{0}-W_{1}}{W_{0}}\times 100$$

### 2.5. Washing after Plasma Treatment

In order to investigate the efficiency of the impurities’ removal by plasma, fabric specimens were subjected to washing at temperatures of 70 °C (hot washing) and 30 °C (cold washing). In this case, the grey single jersey and interlock fabrics were plasma treated with the same parameters, i.e., 30 L/min helium flow rate, 150 W output, 0.4 L/min oxygen flow rate, 1 mm/s treatment time and 3 mm nozzle-to-substrate distance. The plasma-treated knitted and grey fabrics were washed with water (with a liquor-goods-ratio of 50:1) for 15 min at 70 °C (hot washing) and 30 °C (cold washing). Weights of the washed fabrics were then measured (three measurements) and compared.

### 2.6. Scouring after Plasma Treatment

In order to analyse the efficiency of impurities’ removal by plasma and the effect of plasma treatment on the scouring of cotton fabrics, a conventional scouring process was carried out after plasma treatment at a lower temperature, for a shorter duration. The plasma treatment parameters used were a 30 L/m helium flow rate, 150 W output, 0.4 L/min oxygen flow rate, 1 mm/s treatment time and 3 mm nozzle-to-substrate distance. The conventional scouring process was conducted based on a scouring recipe mentioned in Section 2.2.1 for 60 min at 100 °C for 45 min at different temperatures: 100, 80 and 65 °C. After scouring, the specimens were weighed again for comparison.

### 2.7. Wettability of Fabric (Drop Test (Modified AATCC 79-2014))

The wettability of a textile material can be estimated by a wet spot area formed by a liquid drop [21]. In this study, a liquid drop test was conducted on cotton knitted fabrics by using the modified American Association of Textile Chemists and Colorists (AATCC) test method 79-2014 [22]. Fabric specimens of 5 cm × 5 cm in size were used for the drop test. A volume of 20 µL methylene blue aqueous dye solution was added to the fabric specimens using an autopipet at a fixed height of 5 cm, as shown in Figure 2 [23]. The fabric specimen was placed on top of a plastic film in order to avoid the dye droplet being absorbed by other substrates. The completely wet spotted area of the dye droplet, expressed in dispersion size (mm^2^), is defined as the area within the liquid boundary after the fabric is completely dried. The dispersion size in mm^2^ was measured by graph paper. The time for measuring the complete wetting was limited to 10 min and, if complete wetting did not happen within 10 min, the test was terminated and the surface was considered as non-wettable. Three measurements were taken on both sides of the fabric specimens and then averaged. The dispersion size was used for quantifying the wettability of fabric specimens [24].

### 2.8. Yellowness

The yellowness of the fabric specimens, expressed as the yellowness index, was measured according to ASTM E313 [25] by using a reflectance spectrophotometer Datacolor 650 (Datacolor, Hong Kong, China) under illuminant value D65 and a 10° standard observer.

### 2.9. Scanning Electron Microscopy (SEM)

Surface morphology of the fabric was investigated by scanning electron microscopy (SEM, model JSM-6490, JEOL Ltd., Tokyo, Japan). SEM images were captured at magnification of 3000× in order to reveal the surface morphology changes induced by the conventional and plasma treatment (plasma condition: 160 W output power and 0.2, 0.3 and 0.4 L/min oxygen flow rates were used for capturing SEM images).

### 2.10. Fourier-Transform Infrared Spectroscopy-Attenuated Total Reflection (FTIR-ATR)

The surface chemical composition of the fabric specimens was evaluated by Fourier-transform infrared spectroscopy-attenuated total reflection (FTIR-ATR) analysis. The FTIR-ATR analysis was performed with a Perkin Elmer spectrophotometer (Spectrum 100, Perkin Elmer Ltd., Hong Kong, China). The scanning range was 700–4000 cm^−1^ with an average of 64 scans for each fabric specimen. The resolution of scanning was 4 cm^−1^.

### 2.11. X-ray Photoelectron Spectrometer (XPS)

Other than FTIR-ATR analysis, the surface elemental composition of fabric specimens were analysed by an X-ray photoelectron spectrometer (XPS) (Model: Sengyang SKL-12 electron spectrometer, Leybold Heraeus-Sengyang, Sengyang, China), equipped with a VG CLAM 4 MCD electron energy analyser (Leybold Heraeus-Sengyang, Sengyang, China). The X-ray source was a twin-anode (Mg/Al) source from VG (type XR3E2). Non-monochromatic Mg Kα radiation (1253.6 eV) at a current of 15 mA and 10 kV was used. The base pressure within the XPS chamber was 2 × 10^−9^ mbar. Photo-emitted electrons were collected at a takeoff angle of 45°. XPSPEAK 4.1 software (Informer Technologies, Inc.) was used to analyse spectra to obtain elementary content. The relative intensities of C_1*s*_ and O_1*s*_ peaks were measured.

### 2.12. Data Treatment and Analysis

Three specimens were prepared and three measurements were conducted for each evaluation test. The results were averaged with a 95% confidence level in order to have statistically related data for analysis.

## 3. Results and Discussion

### 3.1. Weight-Loss Measurement

**Figure 3:**
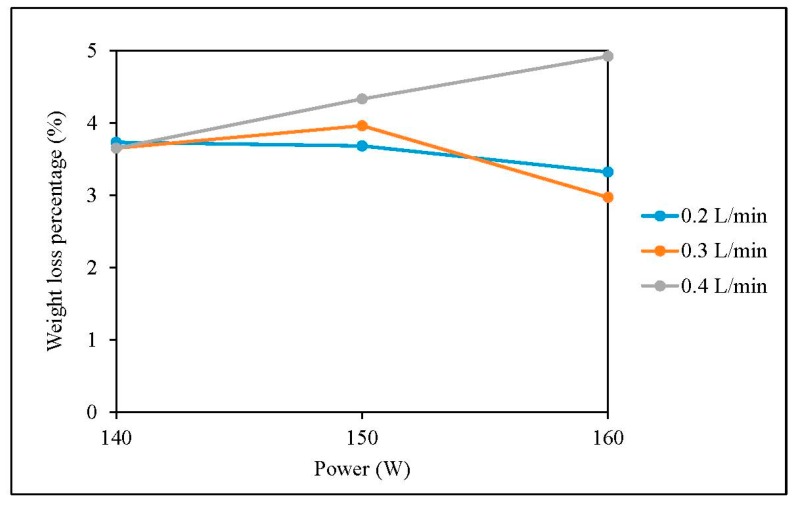
Weight loss percentage of single jersey fabric.

**Figure 4:**
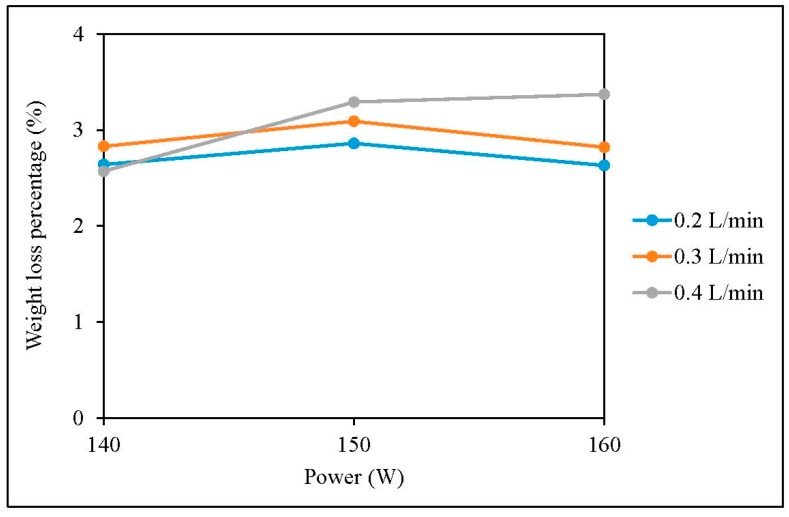
Weight-loss percentages of interlock fabric.

The weight-loss percentages of grey single jersey and interlock fabrics after plasma treatment are shown in Figure 3 and Figure 4 respectively [23].

Figure 3 and Figure 4 show that plasma treatment can reduce the weight of a grey single jersey and interlock fabrics but the degree of weight loss varies, depending on different plasma parameters. The reduction in fabric weight is due to the removal of impurities such as wax and pectin from the fibre surface. Since plasma treatment is a surface-etching process, impurities present on the fibre surface can be broken into small fragments and be etched away by the flow of plasma during the plasma treatment. In order to explore the effectiveness of removing impurities from the grey cotton knitted fabric surface, the surface morphological changes of the grey single jersey and interlock fabric were examined by SEM and the images are shown in Section 3.6.

### 3.2. Washing after Plasma Treatment

After plasma treatment, fabrics were washed with cold (30 °C) and hot (70 °C) water and the weights were calculated and compared. Table 3 and Table 4 show the weight-loss percentage of plasma treated knitted fabrics after cold water washing and hot water washing, respectively. The experimental results reveal that the weights of the plasma treated knitted fabrics decreased further after cold and hot washing. The further decrease in weight is due to breakdown of surface impurities into small fragments, which were etched away from the surface by the flow of plasma (confirmed by the reduction in carbon elemental composition in XPS analysis). Because of the reduction in molecular size, the solubility of the loosened and broken-down impurities increased and then they were easily hydrolysed or dissolved in water [20]. It is obvious that the weight-loss percentages in hot water-washed knitted fabrics are greater than those washed in cold water because high temperature swells the loosened and broken-down impurities in water. Thus, the impurities get hydrolysed and dissolved in hot water effectively, and more impurities can finally be removed.

### 3.3. Scouring after Plasma Treatment

In order to evaluate the efficiency of plasma treatment, plasma-treated single jersey and interlock fabrics were scoured at different temperatures and varying durations; the results are shown in Table 5 and Table 6, respectively [23]. As shown in Table 5 and Table 6, both single jersey and interlock fabrics show that weight-loss percentages of plasma-treated fabrics scoured for 45 min at 100 °C are comparable with grey cotton knitted fabrics treated with conventional scouring parameters. Similar differences in weight-loss percentages of scoured knitted fabrics reveal that a shorter scouring time can be applied after plasma treatment which indicates that the plasma treatment can help save time and energy in the scouring process. Due to the plasma-etching process, impurities on the fibre surface can be broken down into tiny substances which enhances their removal during the scouring process. According to the results in Table 5 and Table 6, it is noted that scouring time can be reduced from 60 min to 45 min if knitted cotton fabrics are plasma pretreated. The shortening of treatment time reduces energy consumption.

### 3.4. Wettability—Dispersion Size

**Figure 5:**
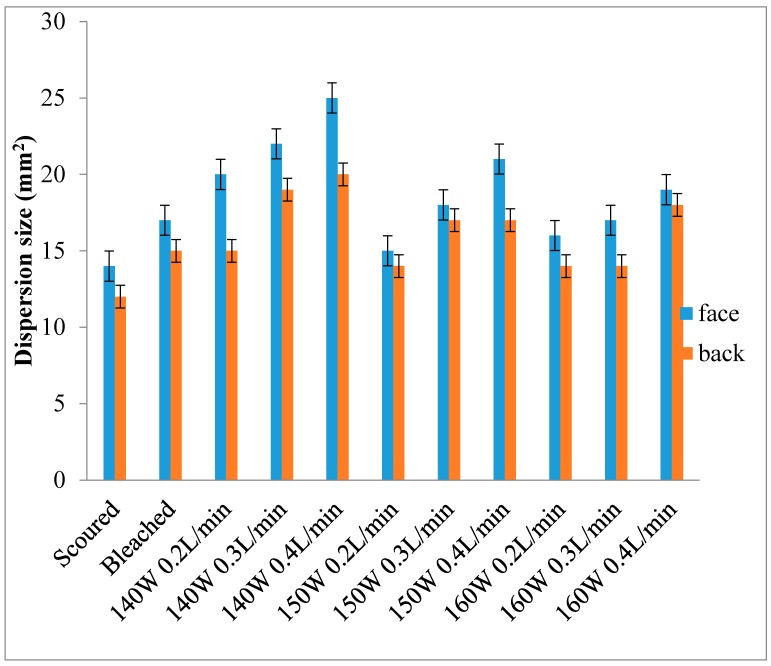
Dispersion size of single jersey fabric.

**Figure 6:**
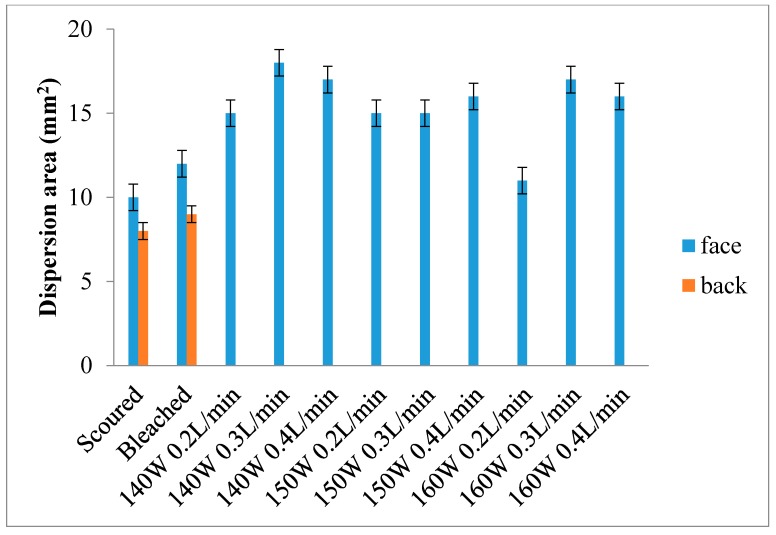
Dispersion size of interlock fabric.

Figure 5 and Figure 6 show the dispersion size of single jersey and interlock fabrics, respectively [25]. In Figure 5 and Figure 6, dispersion size is not there for the grey single jersey and interlock fabrics because the fabric could not be wetted by the dye solution within the time limit of 10 min. Thus, the grey single jersey and interlock fabrics can be considered as non-wettable. A similar non-wettable effect is noted for the back side of plasma-treated interlock fabrics.

Results of single jersey fabrics (Figure 5) show that the wettability of plasma-treated fabrics is significantly higher than that of scoured and bleached fabrics, especially for the fabric treated with 140 W output power. Generally speaking, the higher the output power, the more the kinetic energy-active plasma species carried in the plasma treatment act on the fabric surface. However, if high kinetic energy is carried by plasma species, the high velocity may result in plasmas colliding with each other, easily leading to energy loss. Thus, the amount of active plasma species decreases, resulting in a lesser impact on the fabric surface in the case of 160 W output power [15,16]. In the case of single jersey fabric, wettability is noted on both sides of the fabric but the face side shows a better wetting result than the back side. This phenomenon cannot be observed in interlock fabric because interlock fabric (0.72 mm) is thicker than single jersey fabric (0.46 mm). The plasma species can penetrate through to the back of the single jersey fabric leading to wettability in the fabric’s back side.

In the case of interlock fabrics (Figure 6), the face sides of plasma-treated fabrics show significant enhancement of wettability compared with grey, scoured and bleached fabrics. Generally speaking, interlock fabrics treated with 140 W output power demonstrate better wettability, the reason being the same as in the case of single jersey fabric. Unlike single jersey fabric, the back side of the plasma-treated interlock fabric cannot be wetted by the dye solution, which may be due to its higher thickness [15,16]. Although plasma treatment only affects the fabric surface, penetration power of plasma species, theoretically, the back side may also be affected by the plasma species [15,16]. Since the interlock fabric is much thicker than single jersey fabric, the plasma species cannot not pass through from one side to another side. Therefore, the back side of interlock fabric cannot be affected and wetted.

Single jersey fabric treated with 140 W output power and a 0.4 L/min flow rate and interlock fabric treated with 140 W output power and a 0.3 L/min flow rate give the largest dispersion size on the fabric face. This should help optimise parameters for plasma treatment.

### 3.5. Yellowness

**Figure 7:**
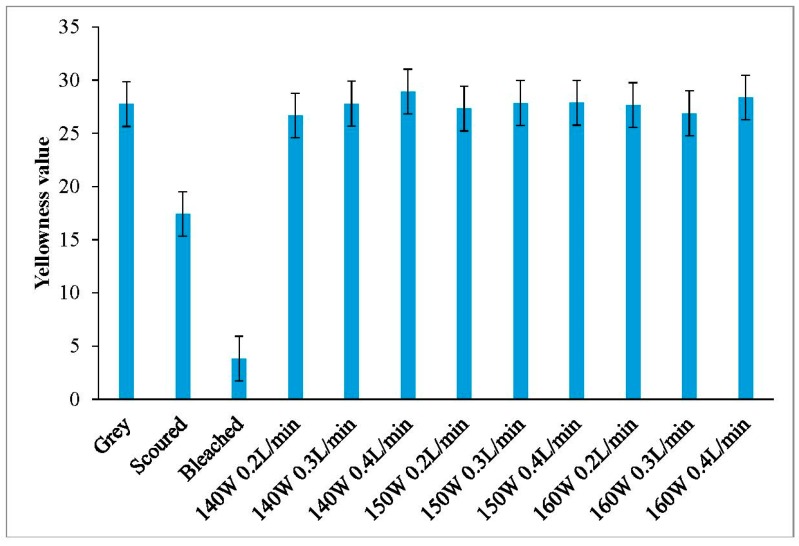
Yellowness values of single jersey fabric.

**Figure 8:**
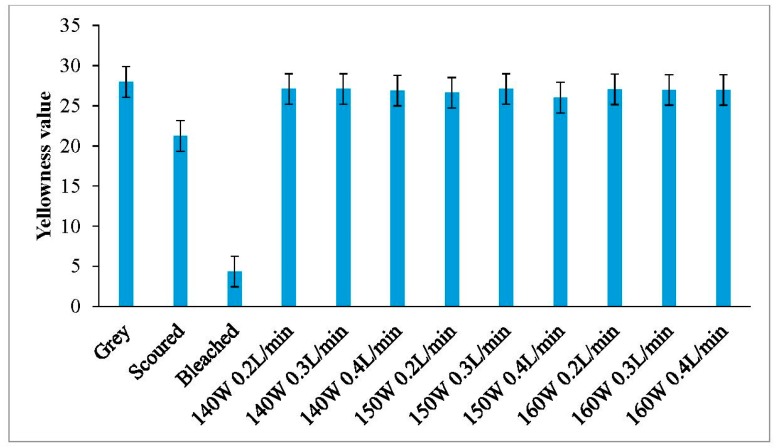
Yellowness values of interlock fabric.

Generally speaking, when a fabric has a high yellowness value, it is yellower. As shown in Figure 7 and Figure 8, the yellowness values of plasma-treated knitted fabrics are similar to the grey fabric. Although plasma treatment is powerful in removing surface impurities from knitted fabrics, a side effect of yellowness may be introduced due to thermal oxidation on the fibre surface [26,27]. Based on the results in Figure 7 and Figure 8, the color of grey cotton knitted fabric is yellowish in nature, but this yellow color does not get further enhanced even after plasma treatment.

### 3.6. Surface Morphology

**Figure 9:**
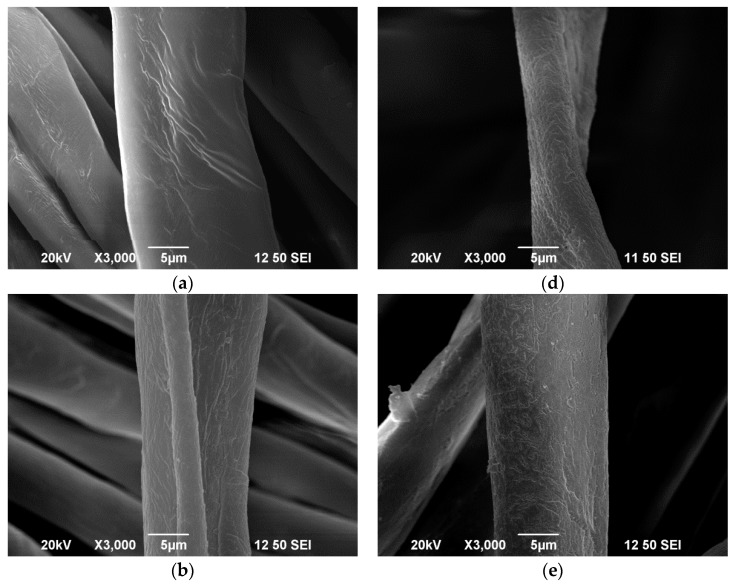

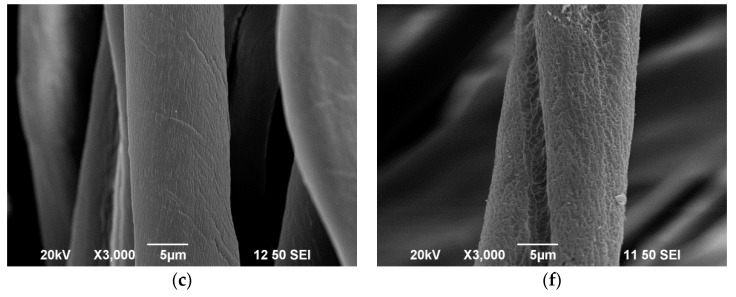
Single jersey fabric: (**a**) grey; (**b**) scoured; (**c**) bleached; and plasma-treated with: (**d**) 160 W 0.2 L/min; (**e**) 160 W 0.3 L/min; and (**f**) 160 W 0.4 L/min.

**Figure 10:**
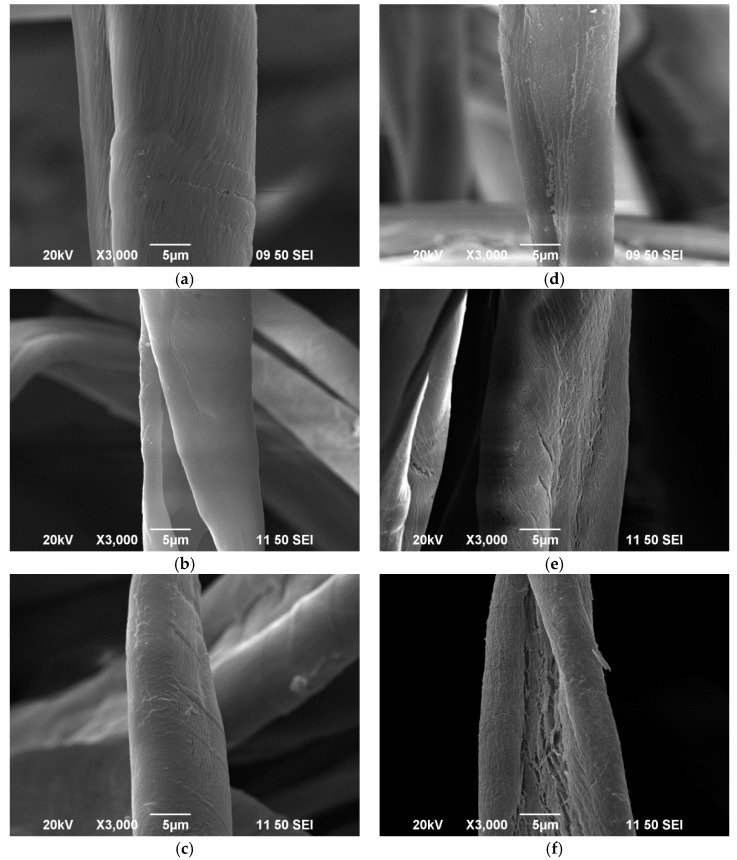
Interlock fabric: (**a**) grey; (**b**) scoured; (**c**) bleached; and plasma-treated with: (**d**) 160 W 0.2 L/min; (**e**) 160 W 0.3 L/min; and (**f**) 160 W 0.4 L/min.

The surface morphology of knitted fabrics is shown in SEM images. Figure 9 and Figure 10 show the surface morphology of single jersey and interlock fabrics under different treatment conditions, respectively.

#### 3.6.1. Effect of Conventional Treatment

SEM images show wax and impurities covering the surface of grey fabrics, both single jersey (Figure 9a) and interlock (Figure 10a). However, a cleaner fibre surface is shown after scouring (single jersey, Figure 9b, and interlock, Figure 10b) and bleaching (single jersey, Figure 10c, and interlock, Figure 10c) processes.

#### 3.6.2. Effect of Plasma Treatment

After plasma treatment, the surface morphology changes and this can be observed clearly in SEM images, as shown in Figure 9d–f (single jersey) and Figure 10d–f (interlock). Based on these SEM images, it can be observed that a large number of microcracks appeared on the fibre surface which reveals that etching occurs. Furthermore, due to the breakdown of pectin and the non-cellulosic molecular chain by the plasma active species during treatment, fragmentation occurs on the fibre surface. In addition, cracks and porous structures are created after plasma treatment due to etching. Those cracks and pores increase the fibre surface area leading to the improved hydrophilicity of the plasma-treated fabrics when compared with grey and scoured knitted fabrics. This improved hydrophilicity after plasma treatment has been evidenced in wettability measurement in Section 3.4.

### 3.7. FTIR-ATR

**Figure 11:**
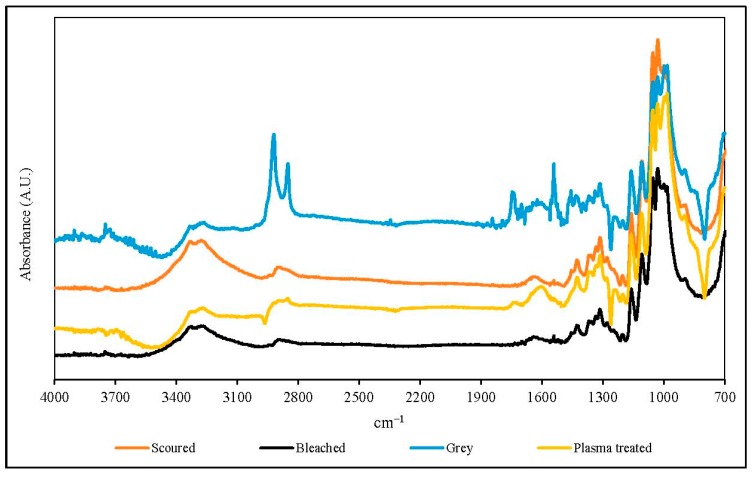
Fourier-transform infrared spectroscopy-attenuated total reflection (FTIR-ATR) spectra of single jersey fabrics (plasma condition: 150 W output power and 0.4 L/min oxygen flow rate).

**Figure 12:**
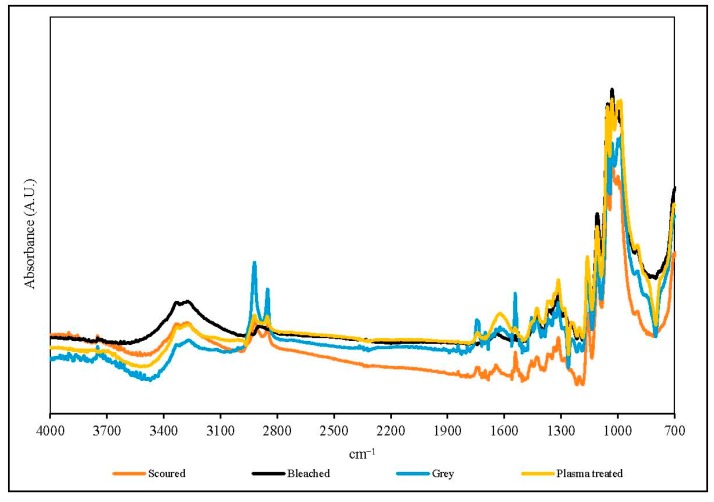
FTIR-ATR spectra of interlock fabrics (plasma condition: 150 W output power and 0.4 L/min oxygen flow rate).

Figure 11 and Figure 12 show the FTIR-ATR spectra of single jersey and interlock fabrics treated under different conditions respectively. The FTIR-ATR analysis can help to study the interactions between the cotton knitted fabrics and the plasma species. The FTIR-ATR spectra of the single jersey and interlock fabrics provide similar results under the same treatment.

The intensities of absorption peaks in the range of 3400–3200 cm^−1^ (O–H stretch) of plasma-treated knitted fabrics are slightly higher than the grey fabric, which confirms an additional O–H stretch has been induced by the oxygen plasmas [5]. The intensities of peaks in the region 1657–1605 cm^−1^ (C=O band) are increased and a small peak appears after plasma treatment, which confirms the oxidation effect on the fabric surface after plasma treatment. After removal of wax by plasma treatment, cotton is oxidised by plasma active species coming from oxygen, and thus a relatively large amount of the C=O band is produced [5,28], as evident from the increased peak intensities of 1657–1605 cm^−1^. Moreover, in the regions of 1330–1000 cm^−1^, higher peaks are observed in plasma-treated knitted fabrics. The regions of 1330–1000 cm^−1^ are related to oxygen-containing groups of the C–O stretch. The increase in the C=O band and C–O stretch confirm that additional polar groups have been induced on to the fibre surface by oxygen plasma. This can enhance the hydrophilicity of the fabric and, as a result, the plasma-treated knitted fabric is more hydrophilic than the grey fabric (Section 3.4).

The peak in the region 2980–2800 cm^−1^ corresponds to asymmetric and symmetric stretching of methylene (–CH_2_–) groups in long alkyl chains, which indicates the presence of wax in the grey fabric [29]. Clearly, this peak disappears after bleaching, but the intensity decreases after scouring when compared with grey fabric. This peak is diminished in single jersey and interlock fabrics also, after plasma treatment, and the effect is more significant in single jersey fabric.

### 3.8. XPS

Table 7 shows elemental composition of single jersey and interlock fabrics treated under different conditions. It can be noted that concentration of C_1*s*_ decreased and that of O_1*s*_ increased after plasma treatment when compared with grey fabrics. However, the decrease in concentration of C_1*s*_ in scoured and bleached fabric is more significant than in plasma-treated fabric because the scouring and bleaching action can penetrate deeper than the plasma action. The decrease in elemental concentration of C_1*s*_ indicates the carbon bonds present on the fabric surface, such as natural impurities, have been broken or removed by plasma treatment. On the other hand, the increase in O_1*s*_ indicates oxygen-containing groups such as C=O, –C=O, O–C–O and O–C=O are formed on the fibre surface after oxygen plasma treatment [12,30]. The etching effect occurred on the fibre surface is evident from the SEM images (Figure 10 and Figure 11).

Furthermore, the decrease in elemental concentration of C_1*s*_ and the increase in elemental concentration of O_1*s*_ affect the O/C ratio. An increase in O/C ratio is noted in plasma-treated knitted fabrics, which is 2 times higher than grey knitted fabric. This significant increase in O/C ratio after plasma treatment leads to enhancement of fabric hydrophilicity and better wettability performance than the grey fabrics.

## 4. Conclusions

This study explores the use of atmospheric pressure plasma for treating grey cotton knitted fabrics (single jersey and interlock). The weight loss and wettability of plasma-treated knitted fabrics were measured and analysed and the results compared with conventionally treated fabrics. Weight loss occurred in plasma-treated fabrics revealing that plasma treatment can help remove impurities present on the fabric surface. The hydrophilicity of grey cotton knitted fabrics was found to be greatly enhanced after plasma treatment. On the other hand, if 100% grey cotton knitted fabrics are pretreated with plasma, duration of the normal scouring process can be reduced. The surface morphological and chemical changes in plasma-treated fabrics were studied and compared with conventionally treated fabrics using SEM, FTIR-ATR and XPS. SEM results revealed that impurities on the surface of knitted fabrics can be removed by plasma treatment. Oxygen-containing groups, confirmed by FTIR-ATR, were induced to the surface of knitted fabrics, which helped improve fabric wettability. The decrease in carbon content, as shown in XPS, revealed the removal of surface impurities. The O/C ratios of plasma-treated knitted fabrics revealed enhancement of fabric hydrophilicity. In addition, the yellowness of plasma-treated grey cotton knitted fabrics did not increase further after plasma treatment.

## Figures and Tables

**Table 1:** Specifications of 100% grey cotton knitted fabrics.

Fabric specification	Single jersey	Interlock
Weight (g/m^2^)	255	385
Yarn count (s)	30	32
Fabric thickness (mm)	0.46	0.72
Fabric density (wales per inch × courses per inch)	48 × 31	42 × 35

**Table 2:** Parameters used in plasma treatment.

Plasma process parameters	Values		
Flow rate of helium (liter per minute, L/min)	30		
Flow rate of oxygen (liter per minute, L/min)	0.2	0.3	0.4
Treatment time (mm/s)	1		
Output power (W)	140	150	160
Nozzle-to-substrate distance (mm)	3		

**Table 3:** Weight loss of plasma-treated knitted fabric with cold (30 °C) washing (plasma condition: 150 W output power and 0.4 L/min flow rate).

Fabric type	Weight after plasma treatment (g)	Weight after cold washing (g)	Weight difference (g)	Weight loss percentage (%)
Single jersey	5.72	5.58	0.14	2.45
Interlock	8.06	7.78	0.28	3.47

**Table 4:** Weight loss of plasma-treated knitted fabric with hot (70 °C) washing (plasma condition: 150 W output power and 0.4 L/min flow rate).

Fabric type	Weight after plasma treatment (g)	Weight after hot washing (g)	Weight difference (g)	Weight loss percentage (%)
Single jersey	5.78	5.60	0.18	3.11
Interlock	8.12	7.82	0.30	3.69

**Table 5:** Weight-loss difference (WLC) between plasma-treated and scoured single jersey fabric (plasma condition: 150 W output power and 0.4 L/min flow rate).

Grey (g)	After plasma treatment (g)	Scouring duration (min)	Scouring temperature (°C)	Scoured (g)	Weight loss percentage (%)	WLC when compared with normal scouring (%) *
6.06	–	60	100	5.56	8.25	0
5.92	5.66	60	100	5.44	8.11	−0.14
5.84	5.60	45	100	5.40	7.53	−0.72
5.96	5.70	45	80	5.54	7.05	−1.20
5.96	5.72	45	65	5.58	6.38	−1.87

* Negative value refers to worse scouring efficiency when compared with normal scouring process.

**Table 6:** Weight loss difference (WLC) between plasma-treated and scoured interlock fabric (plasma condition: 150 W output power and 0.4 L/min flow rate).

Grey (g)	After plasma treatment (g)	Scouring duration (min)	Scouring temperature (°C)	Scoured (g)	Weight loss percentage (%)	WLC when compared with normal scouring (%) *
8.54	–	60	100	7.92	7.26	0
8.70	8.38	60	100	8.10	6.90	−0.36
8.70	8.34	45	100	8.10	6.90	−0.36
8.74	8.38	45	80	8.16	6.64	−0.62
8.82	8.44	45	65	8.24	6.58	−0.68

* Negative value refers to worse scouring efficiency when compared with normal scouring process.

**Table 7:** Surface elemental analysis and atomic ratios of knitted fabrics (plasma condition: 150 W output power and 0.4 L/min flow rate).

**Element concentration (%)**				
**Fabric sample**	**C_1*s*_**		**O_1*s*_**	
	**Single jersey**	**Interlock**	**Single jersey**	**Interlock**
Grey	0.75	0.75	0.25	0.25
Scoured	0.47	0.49	0.53	0.51
Bleached	0.37	0.42	0.63	0.58
Plasma	0.71	0.59	0.29	0.41
**Atomic Ratio (O/C)**				
**Fabric sample**	**Single jersey**		**Interlock**	
Grey	0.33		0.33	
Scoured	1.13		1.04	
Bleached	1.70		1.38	
Plasma	0.41		0.69	
